# Reporting of Systematic Reviews: The Challenge of Genetic Association Studies

**DOI:** 10.1371/journal.pmed.0040211

**Published:** 2007-06-26

**Authors:** Muin J Khoury, Julian Little, Julian Higgins, John P. A Ioannidis, Marta Gwinn

We applaud PLoS editors for their commitment to publishing high-quality systematic reviews (SRs) [1]. Moher et al. [2] clearly documented the inconsistent quality of reporting of SRs. With more than 2,500 SRs published every year, low-quality or outdated reviews may mislead researchers, providers, and policy makers. The situation could be improved if more evidence-based reporting guidelines were agreed upon, developed, and adhered to.

The growing field of genetic associations (GAs) illustrates the urgent need for transparent SRs and meta-analyses. Already, thousands of articles on GAs have been published, and the application of high-throughput genotyping methods may exponentially increase the number of reported associations [3]. Selective reporting of large numbers of false-positive associations could undermine the field and interfere with our ability to translate advances in genomics into clinical practice.

To address these problems, the Human Genome Epidemiology Network (HuGENet) was started as a global collaboration to strengthen methods of analysis and reporting of GAs and to develop a reliable knowledge base on the association between genetic variation and human diseases [4]. Between 2001 and 2006, the HuGENet online database assembled more than 25,000 published articles on GAs and more than 500 systematic reviews of GAs. Nevertheless, there are large inconsistencies in the quality of genetic association studies [5] and in the reporting of SRs of such associations [6]. In collaboration with several journals, HuGENet promotes the publication of transparently reported SRs of gene–disease associations [4]. More than 50 HuGE reviews have been published over the past six years.

After several HuGENet workshops bringing together researchers from different fields and journal editors, the first edition of a HuGENet handbook, modeled in part after the Cochrane handbook of systematic reviews, was published on the Canadian HuGENet Web site [7]. The handbook describes methodological issues and outlines steps in conducting such reviews, including the need for a detailed protocol. It also discusses meta-analysis methods. We strongly encourage researchers interested in conducting systematic reviews of GAs to consult the HuGENet handbook, and adopt transparent protocols. Retrospective SRs of published data have limitations, even when properly conducted. Investigators can advance the field of human genome epidemiology by conducting prospective meta-analyses and large collaborative analyses through international consortia. HuGENet has created a Network of Investigator Networks to help the growth of such initiatives [8].
